# Species-specific differences in synaptic transmission and plasticity

**DOI:** 10.1038/s41598-020-73547-6

**Published:** 2020-10-06

**Authors:** Prateep Beed, Saikat Ray, Laura Moreno Velasquez, Alexander Stumpf, Daniel Parthier, Aarti Swaminathan, Noam Nitzan, Jörg Breustedt, Liora Las, Michael Brecht, Dietmar Schmitz

**Affiliations:** 1 Neuroscience Research Center, Charité-Universitätsmedizin Berlin, Berlin, Germany; 2 Berlin Institute of Health, 10178 Berlin, Germany; 3 Bernstein Center for Computational Neuroscience, Humboldt University of Berlin, Philippstr. 13, Haus 6, 10115 Berlin, Germany; 4 Department of Neurobiology, Weizmann Institute of Science, 76100 Rehovot, Israel; 5 German Center for Neurodegenerative Diseases (DZNE), 10117 Berlin, Germany; 6 Cluster of Excellence NeuroCure, 10117 Berlin, Germany; 7 Einstein Center for Neurosciences Berlin, 10117 Berlin, Germany

**Keywords:** Synaptic plasticity, Synaptic transmission

## Abstract

Synaptic transmission and plasticity in the hippocampus are integral factors in learning and memory. While there has been intense investigation of these critical mechanisms in the brain of rodents, we lack a broader understanding of the generality of these processes across species. We investigated one of the smallest animals with conserved hippocampal macroanatomy—the Etruscan shrew, and found that while synaptic properties and plasticity in CA1 Schaffer collateral synapses were similar to mice, CA3 mossy fiber synapses showed striking differences in synaptic plasticity between shrews and mice. Shrew mossy fibers have lower long term plasticity compared to mice. Short term plasticity and the expression of a key protein involved in it, synaptotagmin 7 were also markedly lower at the mossy fibers in shrews than in mice. We also observed similar lower expression of synaptotagmin 7 in the mossy fibers of bats that are evolutionarily closer to shrews than mice. Species specific differences in synaptic plasticity and the key molecules regulating it, highlight the evolutionary divergence of neuronal circuit functions.

## Introduction

The Etruscan shrew *(Suncus etruscus)* is the smallest terrestrial mammal, with a full-grown adult weighing ~ 2 g and having a brain volume of ~ 60 mm^3^^1,2^. It is approximately 15 times smaller in body size and has a brain ~ 7 times smaller than a lab mouse. They hunt for their food, and prey on insects like crickets—consuming multiple times their body weight every day, primarily using somatosensory input from their whiskers^3^ to guide hunting. Despite the minutely sized brain, the overall layout of the brain is rather similar to other mammalian brains, with a 6-layered cortex^1,2^ and conserved genetic and architectural features in the neocortex and hippocampal formation^1,2,4^.

The hippocampus is a key structure in learning and memory in the central nervous system, and activity dependent changes in synaptic strength are thought to be the underlying cellular correlate^5–9^. Information to the hippocampus is routed from the neocortex through the evolutionarily conserved trisynaptic pathway^10^. The mossy fiber and the Schaffer collateral synapses are among the most investigated synapses in neuroscience—though most investigation has been limited to rodent studies. As the Etruscan shrew is one of the smallest animals with clearly defined hippocampal substructures, and features these two synapses, we compared the anatomy and physiology of these two synapses in the Etruscan shrew and in mice.

**Figure 1:**
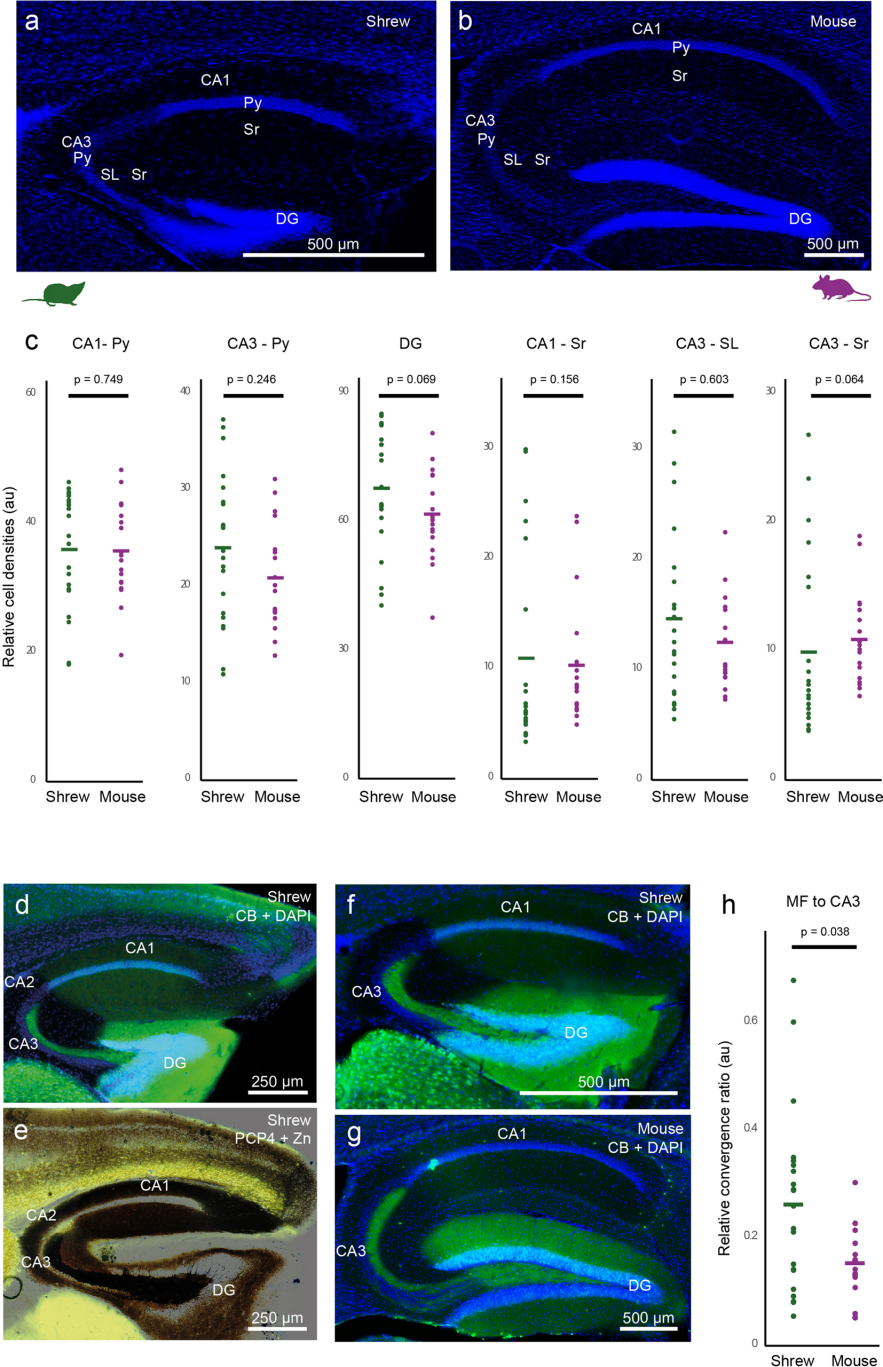
Macroanatomy of the Etruscan shrew hippocampus is similar to mice. Sagittal sections stained for DAPI showing the hippocampus of (**a**) shrew and (**b**) mice indicating the different subregions of the hippocampus—dentate gyrus (DG), CA1, CA3 and the pyramidal (Py), Stratum Radiatum (Sr) and Stratum Lucidum (SL) layers. (**c**) Relative cell densities in different hippocampal subregions do not differ between shrews (green) and mice (purple), indicating conserved cytoarchitectonic layout (p-values based on Mann–Whitney two tailed test.). (**d**,**f**) Saggital section of an Etruscan shrew hippocampus labelled for calbindin (CB, green) and DAPI (blue) indicating the different hippocampal subregions—CA1, CA2, CA3, dentate gyrus (DG) and mossy fibers (mf). (**e**) Mossy fibers (mf) visualized by the presence of synaptic zinc (brown) in a sagittal section of an Etruscan shrew brain, and CA2 labelled by the presence of the protein PCP4 (yellow) show that the mossy fibers are present in CA3 and terminate at the CA2 region. (**g**) Sagittal section of a mouse, marked same as (**f**). (**h**) Relative convergence ratios between mossy fiber intensities and cell density reveal a higher mossy fiber to CA3 pyramidal cell convergence factor in shrews than in mice.

We first investigated the architecture of the hippocampus of the shrew, to determine if tissue size and space constraints affect the structural layout of hippocampal circuits. The overall layout of the hippocampus in shrews is similar to other mammalian species, with classical subfields like the dentate gyrus, CA1, CA2 and CA3 (Fig. 1). These areas can be easily distinguished by similar cytoarchitecture (Fig. 1a,b), cell densities (Fig. 1c) and consistent histochemical and immunohistochemical features (Fig. 1d–g) between shrews and mice. Histochemistry and immunohistochemistry also reveals that the major fiber pathways like the mossy fiber pathway are also conserved, with the mossy fibers in CA3 being enriched in synaptic zinc (Fig. 1e, brown) and the calcium binding protein calbindin (CB, green; Fig. 1d,f). The mossy fibers extend through the entire CA3 region in the stratum lucidum layer and terminate at the CA2 region—which has neurons expressing the protein purkinje cell protein 4 (PCP4, yellow; Fig. 1e). However, the anatomy of mossy fibers in shrews indicates subtle differences with shrews having a slightly higher relative convergence ratio of mossy fiber inputs (Fig. 1f–h) in CA3 than mice.

We then explored if there were any corresponding differences in synaptic transmission and plasticity in the mossy fiber pathway. We recorded mossy fiber fEPSPs from sagittal slices of shrews using the same solutions and slicing procedure as used for mice (Fig. S1a). Mossy fiber inputs showed fEPSP/ fiber volley (FV) ratio similar to that in mice as well as its sensitivity to the agonist for the group II metabotropic glutamate receptors, dcg iv (Fig. S1b–d, Table S1). Therefore, synaptic transmission at the shrew mossy fiber is similar to that in mice.

**Figure 2:**
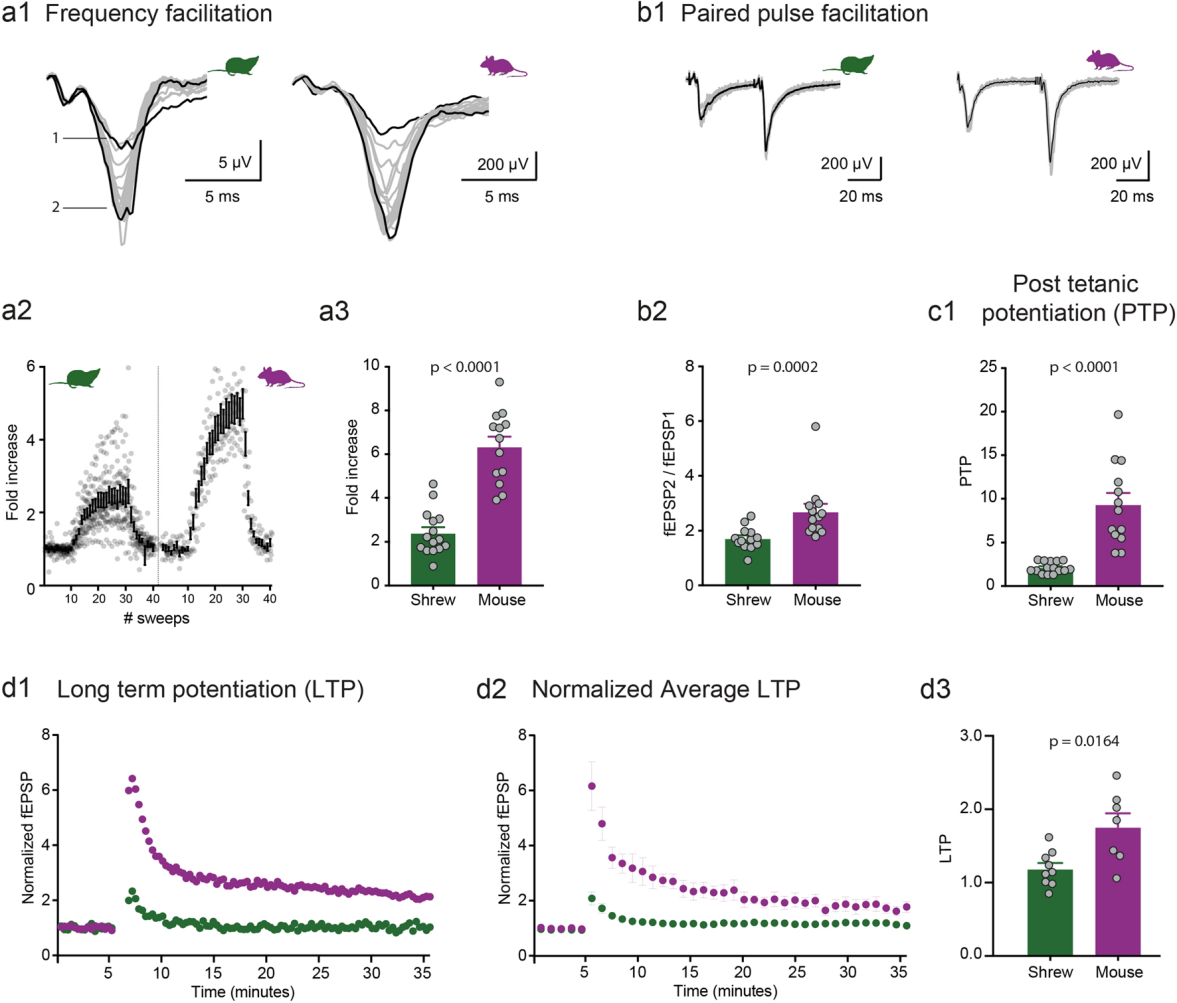
Low synaptic plasticity at mossy fiber synapse of the Etruscan shrew. Different short-term plasticity parameters are quantified and compared at the mossy fiber synapse between shrews (green) and mice (purple). (**a1**) 1 Hz frequency facilitation in shrews versus mice. (**a2**) Lower frequency facilitation in shrews as compared to mice in (**a3**). (**b1**) Paired pulse facilitation with 50 ms inter pulse interval is also lower in shrews as compared to mice in (**b2**). (**c1**) Following 4 × tetanic stimulation, the post-tetanic potentiation was also compared between shrews and mice. Several-folds lower potentiation in shrews as compared to mice. Long-term plasticity at the mossy fiber synapse between shrews (green) and mice (purple). (**d1**) Single example of mossy fiber LTP in shrew (green) versus mouse (purple). (**d2**) Average LTP data shows lower levels of mossy fiber LTP in shrews as compared to mice. (**d3**) Shrews show lower levels of mossy fiber LTP as compared to mice.

The plasticity at the mossy fiber synapse is primarily presynaptic^11,12^. Most of the unique parameters that classifies mossy fibers as detonator synapses are due to its short-term plasticity (STP) such as 1 Hz frequency facilitation, paired-pulse ratio and post-tetanic potentiation. We investigated these short-term plasticity features at the mossy fiber synapse in shrews and compared it to mice. Surprisingly, we found that shrew mossy fiber synapses are much less plastic than those in mice, even though the relative convergence ratio of mossy fiber inputs in CA3 is higher in shrews than in mice (Fig. 1h). Frequency facilitation was measured by stimulating the mossy fiber inputs twenty times (Fig. 2a1,a2, sweeps 10–30) at 1 Hz. The ratio of the 30th (marked as 2 in Fig. 2a1) to the 10th (marked as 1 in Fig. 2a1) sweep was calculated for both shrews and mice to determine the frequency facilitation: we found that shrews had a much lower facilitation in comparison with mice (Fig. 2a3 shrews: 2.41 ± 0.26 fold increase, n = 15 recordings; mice: 6.34 ± 0.46 fold increase, n = 13 recordings, p < 0.0001, Mann–Whitney unpaired test). Paired pulse ratio (PPR) was calculated as the peak of second EPSP to the first EPSP which had an inter-pulse interval of 50 ms (Fig. 2b1). Paired pulse ratio was also significantly lower: shrews—1.72 ± 0.12, n = 13 recordings; mice—2.69 ± 0.29, n = 13 recordings, p = 0.0002, Mann–Whitney unpaired test (Fig. 2b2). Lastly to determine the post-tetanic potentiation (PTP) we stimulated 4 × at an interval of 20 s and each stimulus had 125 pulses at 25 Hz. PTP was determined as the average of the 3 sweeps following the repetitive stimulation. Here, we found a dramatic difference in the amount of PTP, with shrews having a much lower PTP than mice—shrews: 2.12 ± 0.17, n = 15 recordings; mice: 9.34 ± 1.32, n = 13 recordings, p < 0.0001, Mann–Whitney unpaired test (Fig. 2c1). When lowering extracellular Ca concentration from 2.5 to 1.5 mM, we observed the predicted change in fEPSP/FV ratio; however, shrews showed only moderate changes in PPR (paired-pulse facilitation, Fig. S2a–c, Table S1).

Finally, we analyzed LTP at this particular synapse. Following a stable baseline of synaptic transmission, we induced plasticity by using tetanic stimuli as mentioned above—a protocol which has been successfully used in many different preparations^27^. At this particular synapse there is a significantly lower level of LTP in shrews as compared to mice (Fig. 2d1–d3). Although lower than mice, mossy fiber LTP in shrews was significantly different from the mean normalized value of 1 (one-sample t-test, p = 0.045).

**Figure 3:**
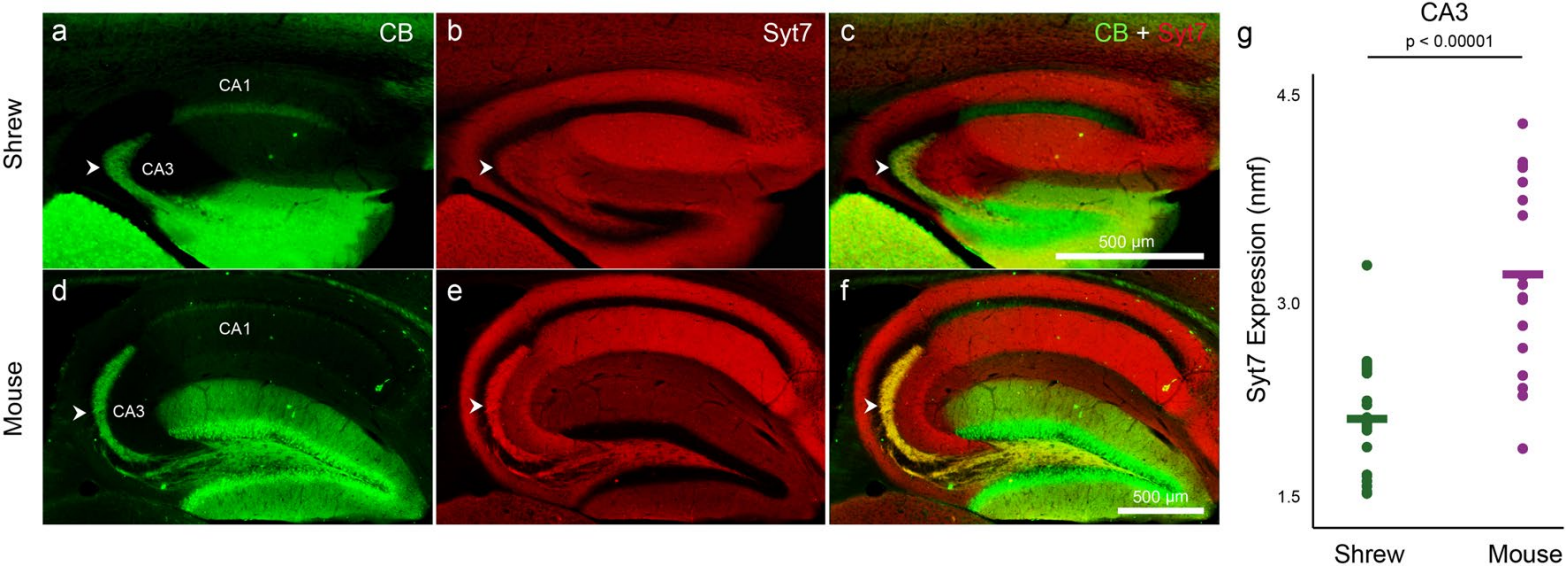
Etruscan shrew mossy fibers have low synaptotagmin 7. Hippocampal mossy fibers (white arrowheads) are labeled with calbindin (green) in shrews (**a**) and mice (**d**). The same sections co-labeled with synaptotagmin 7 (**b**,**e**; Syt7, red) and overlaid in (**c**) and (**f**) respectively, show low Syt7 expression in the shrew CA3-mossy fibers (**b**,**c**) but not in mice (**e**,**f**). Note the yellow colour of the mossy fibers in (**f**) but not in (**c**) due to the lack of Syt7 in shrews. Quantification of normalized mean fluorescence levels of Syt7 in shrews and mice (**g**) indicates that the CA3-mossy fibers in shrews have lower Syt7 expression than mice. Scale bar in (**c**) and (**f**) also applies to (**a**,**b**) and (**c**,**d**) respectively.

**Figure 4:**
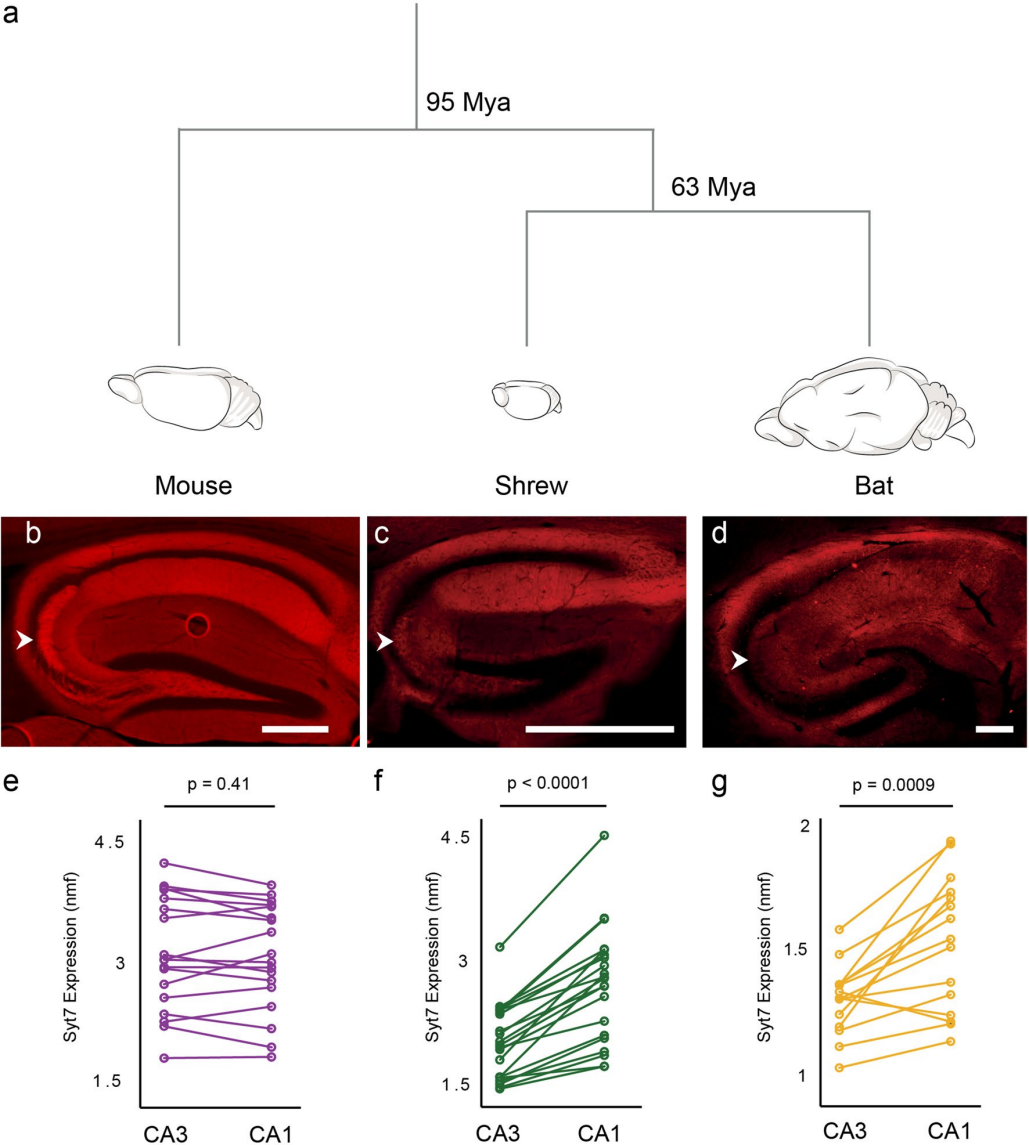
Synaptotagmin 7 distribution in CA3 and CA1 of mice, shrews and bats. (**a**) Evolutionary time-line between mice, shrews and bats shows bats are closer relatives of shrews than mice ^4,26^. (**b**–**d**) Syt 7 expression in the hippocampus of mice in (**b**), shrews in (**c**) and bats in (**d**). White arrowheads indicate the mossy fibers. (**e–f**) Quantification of Syt 7 between CA3 and CA1 area of mice in (**e**), shrews in (**f**) and bats in (**g**). Expression patterns of Syt 7 are similar between bats and shrews compared to mice which correlates well with their phylogeny. Scale bars 500 μm.

The calcium sensing protein, synaptotagmin 7 (Syt7) plays an important and reversible role in mediating short term plasticity^13^. To investigate if this molecular substrate contributes to the underlying difference between mice and shrews, we performed immunohistochemical investigation for the presence Syt7. Low levels of Syt7 leads to markedly lower plasticity potential at synapses like the mossy fibers, where the presynaptic side dominates^13^. Immunohistochemical processing of hippocampal sections with calbindin in shrews (Fig. 3a) and mice (Fig. 3d) marks the mossy fibers. Co-processing the same sections for Syt7 (Fig. 3b,e) shows strikingly lower levels of Syt7 expression in the shrew compared to mice mossy fibers (overlays in Fig. 3c,f,g, Table S1), despite a higher convergence ratio of mossy fibers from the dentate gyrus to the CA3 in shrews (Fig. 1h, Table S1). This posits Syt7 as a strong candidate for the observed differences in short-term plasticity at the mossy fiber between shrews and mice. To assess the evolutionary basis of these species differences in synaptotagmin 7 expression, and thus perhaps short term plasticity, we extended our anatomical studies to Egyptian fruit bats (*Rousettus aegyptiacus*), which genomic studies have indicated are evolutionarily closer to shrews than mice^4,26^ (Fig. 4a). Indeed, we saw that Syt7 expression in the bat and shrew hippocampus followed a similar pattern—with comparatively lower expression of Syt7 in CA3 than in CA1, than that observed in mice (Fig. 4b–g, Table S1).

**Figure 5:**
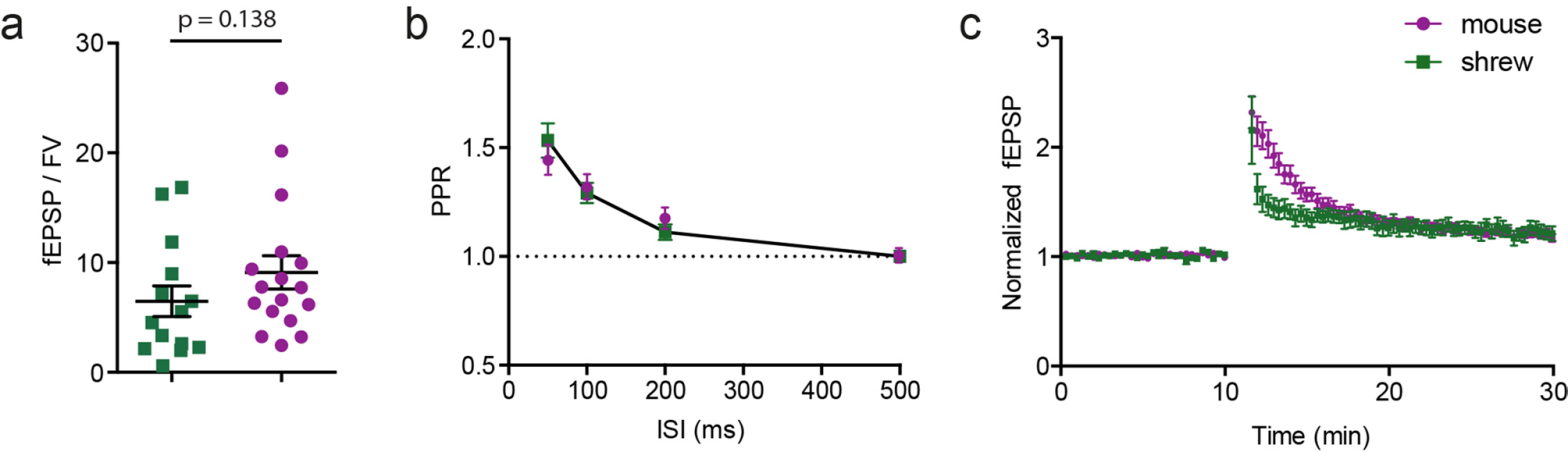
CA1 Schaffer collateral synapse in the shrew and mouse. (**a**) Field EPSP to presynaptic fiber volley ratio. (**b**) Paired pulse ration at 4 different time intervals of 50 ms, 100 ms, 200 ms and 500 ms. (**c**) LTP at this synapse is comparable between shrew and mouse.

We then assessed if the differences in synaptic transmission and plasticity in shrews were limited to CA3 mossy fibers, or extended also to the CA1 Schaffer collateral pathway. We therefore performed both field (with synaptic stimulation of the Schaffer collateral synapses) and whole cell recordings of shrew CA1 pyramidal cells to investigate the properties of synaptic transmission, plasticity and single cell morphological and electrophysiogical features. We found no differences in input/output behaviour as well as in short-term and long-term plasticity in area CA1 of the hippocampus of the shrews (Fig. 5a–c) compared to that observed in mice^28^. We also investigated the basic cellular physiology of CA1 pyramidal neurons by performing whole-cell patch clamp recordings (Fig. S3). We investigated both anatomical details (Fig. S3a,b,c1,c2) and intrinsic cellular properties. Intrinsic properties such as firing pattern and resting membrane potential (Fig. S3d1), input resistance (Fig. S3d2), action potential threshold (Fig. S3d3), action potential amplitude (Fig. S3d4) and action potential FWHM (Fig. S3d5) were similar to the values observed in mice^14^. Similarly, single cell anatomical structures indicate no apparent difference to those reported in mice^14^. Overall this indicated that in shrews the basic properties of cellular architecture and physiology, synaptic transmission and plasticity are largely similar to those observed in well investigated species like mice. However, specific changes in molecular architecture and correlated changes in plasticity at the CA3 mossy fiber synapse points towards species specific adaptation of neural microcircuits.

The mossy fiber synapse in mice has been postulated to work as a detonator synapse^15,16^, with synaptic facilitation due to short term plasticity allowing the synapse to go into a detonation mode, where one spike from a presynaptic granule cell would be sufficient to induce a postsynaptic spike in the postsynaptic CA3 pyramidal neurons. However, shrews show low STP, and based on anatomical similarities, we predict that bats might also have similarly low levels of short-term plasticity. Thus the feasibility of the mossy fiber synapse to act as a detonator synapse might be limited to certain species and perhaps not a general phenomenon.

The importance in learning and memory of the hippocampus has been thoroughly investigated, and hippocampal LTP in general has been observed to be important in the process of spatial learning and memory. However, the investigation of the behavioural impact of mossy fiber synaptic transmission and LTP has not been conclusive, and has produced contradictory findings, with some studies finding that the lack or alterations of mossy fiber transmission results in impaired memory^17–19^, while others indicate there are no such effects^20,21^. However, even though shrews have comparatively lower mossy fiber LTP than mice, they can show spatial memory and hoard food stashes both close and away from their nests^22^ and learn new strategies for hunting^3^. This seems to indicate that plasticity at the mossy fiber synapse might only play a limited role in spatial memory—however its involvement might vary in other forms of episodic-like memories such as contextual memory formation.

In summary, we show that plasticity at the mossy fiber synapse in shrews is markedly distinct—with several fold lower levels of short term plasticity and long term potentiation than that observed in mice. The lower levels of expression of the protein synaptotagmin 7 in shrew mossy fibers might contribute to the physiological differences observed in plasticity. Our findings suggest that while the basic layout of classical circuits like the mossy fiber pathway might be conserved across different mammals, specific genetic differences among them can result in distinct physiology of these circuits and question the functional and behavioural impact of plasticity at the hippocampal mossy fiber synapse.

## Methods

Animal husbandry and experimental interventions were performed in accordance with the German Animal Welfare Act and European Council Directive 86/609/EEC regarding the protection of animals used for experimental and other scientific purposes. All experimental procedures and maintenance of mice were conducted in accordance with permission from local regulatory authorities (Berlin Landesamt für Gesundheit und Soziales, permit T0100/03). All experimental procedures and maintenance of shrews were conducted in accordance with permission from local regulatory authorities (Berlin Landesamt für Gesundheit und Soziales, permits T0160/14 and T0078/16). Bat brains were obtained from experimental procedures approved by the Institutional Animal Care and Use Committee of the Weizmann Institute of Science.

### Electrophysiology

Slice preparation and electrophysiogical recordings were done as described before^28,29^.

### Slice preparation

Shrews and mice of both sexes (2–3 months old) were anesthetized with isofluorane and decapitated. The brain was quickly removed and chilled in ice-cold sucrose-artificial cerebrospinal fluid (sACSF) containing (in mM): 50 NaCl, 25 NaHCO_3_, 10 glucose, 150 sucrose, 2.5 KCl, 1 NaH_2_PO_4_, 0.5 CaCl_2_, and 7 MgCl_2_ for Mossy fiber recordings and 87 NaCl, 26 NaHCO_3_, 10 glucose, 50 sucrose, 2.5 KCl, 1.25 NaH_2_PO_4_, 0.5 CaCl_2_, and 3 MgCl_2_ for Schaffer collateral recordings. All solutions were saturated with 95% O2 (vol/vol) and 5% CO2 (vol/vol), pH 7.4.

Slices (400 μm, sagittal) were cut with a Leica VT1200S microtome (Wetzlar, Germany) and stored submerged in sACSF for 30 min at 35 °C and subsequently stored in ACSF containing (in mM): 119 NaCl, 26 NaHCO_3_, 10 glucose, 2.5 KCl, 1 NaH_2_PO_4_, 2.5 CaCl_2_ and 1.3 MgCl_2_ saturated with 95% O2 (vol/vol) 5% CO_2_ (vol/vol), pH 7.4, at RT. Experiments were started 1–6 h after the preparation.

### Electrophysiological recordings

Electrophysiogical recordings were done as described before^28,29^. In brief, slices were placed in a recording chamber continuously superfused with ACSF at RT at a rate of 2.5 ml/min. fEPSPs were evoked by electrical stimulation with patch pipettes filled with ACSF. fEPSPs were recorded with a low-resistance patch-pipette filled with ACSF. Recordings were performed with a MultiClamp 700B amplifier. Signals were filtered at 2 kHz and digitized (BNC-2090; National Instruments Germany GmbH) at 10–20 kHz. IGOR Pro software was used for signal acquisition (WaveMetrics, Inc.).

For Mossy fiber recordings, stimulation electrodes were placed in the granule cell layer or in the hilus region. Mossy fiber origin of recorded signals was verified by frequency facilitation and a reduction of 80% of the responses upon DCGIV (1 µM; Tocris) application at the end of each experiment. fEPSPs in area CA1 were recorded in stratum radiatum after stimulation of the Schaffer collaterals. fEPSP magnitude was determined by analyzing ± 2 ms of the amplitude peak. Data were analyzed with the Igor plug-in NeuroMatic (neuromatic.thinkrandom.com) software. Statistical analysis was performed with Prism 6 (GraphPad Software).

### Anatomy

#### Brain tissue preparation

Brain tissue preparation were done as described before^23–25^. In brief, male and female mice, Etruscan shrews and Egyptian fruit bats (n = 20 mice, 20 shrews, 5 bats) were used in the study.

Animals were anaesthetized by isoflurane, and then euthanized by an intraperitoneal injection of 20% urethane. They were then perfused transcardially with first 0.9% phosphate buffered saline solution, followed by 4% formaldehyde, from paraformaldehyde, in 0.1 M phosphate buffer (PFA). Subsequently, brains were removed from the skull and postfixed in PFA overnight. Brains were then transferred to 10% sucrose solution for one night and subsequently immersed in 30% sucrose solution for at least one night for cryoprotection. The brains were embedded in Jung Tissue Freezing Medium (Leica Microsystems Nussloch, Germany), and subsequently mounted on the freezing microtome (Leica 2035 Biocut) to obtain 20–60 μm thick sagittal sections or tangential sections parallel to the pia.

Sagittal sections of the hippocampus were obtained by first separating the two hemispheres. The hemisphere was then positioned with the medial surface of the brain being attached to the block face of the microtome to obtain sections.

#### Histochemistry and immunohistochemistry

Hisotchemistry for visualization of synaptic zinc was performed as described previously^23^. In brief, sections were exposed to a solution containing gum arabic, citrate buffer, hydroquinone and silver lactate for 60–120 min, in the dark at room temperature. Development of reaction products was checked under a microscope and terminated by rinsing the sections in 0.01 M PB and, subsequently, several times in 0.1 M PB.

Immunohistochemical stainings were performed according to standard procedures and as described previously^24,25^. Briefly, brain sections were pre-incubated in a blocking solution containing 0.1 M PBS, 2% Bovine Serum Albumin (BSA) and 0.5% Triton X-100 (PBS-X) for an hour at room temperature (RT). Following this, primary antibodies were diluted in a solution containing PBS-X and 1% BSA. Primary antibodies against the calcium binding proteins Calbindin (Swant: CB300, CB 38; 1:5000), the calmodulin binding protein Purkinje cell protein 4 (Sigma: HPA005792; 1:200) and the calcium sensing protein Synaptotagmin 7 (Synaptic Systems: 105173; 1:200) were used. Incubations with primary antibodies were allowed to proceed for at least 24 h under mild shaking at 4 °C in free-floating sections. Incubations with primary antibodies were followed by detection with secondary antibodies coupled to different fluorophores (Alexa 488, 546 and 633; Invitrogen). Secondary antibodies were diluted (1:500) in PBS-X and the reaction allowed to proceed for two hours in the dark at RT. For multiple antibody labeling, antibodies raised in different host species were used. For visualizing cell nuclei, sections were counterstained with DAPI (Molecular Probes: R37606). After the staining procedure, sections were mounted on gelatin coated glass slides with Vectashield mounting medium (Vectorlabs: H-1000).

#### Image acquisition

Similar to our previous studies^23–25^ an Olympus BX51 microscope (Olympus, Shinjuku Tokyo, Japan) equipped with a motorized stage (LUDL Electronics, Hawthorne NY, USA) and a z-encoder (Heidenhain, Shaumburg IL, USA), was used for bright field microscopy. Images were captured using a MBF CX9000 (Optronics, Goleta CA, USA) camera using Neurolucida or StereoInvestigator (MBF Bioscience, Williston VT, USA). A Leica DM5500B epifluorescence microscope with a Leica DFC345 FX camera (Leica Microsystems, Mannheim, Germany) was used to image the immunofluorescent sections. Alexa fluorophores were excited using the appropriate filters (Alexa 350—A4, Alexa 488—L5, Alexa 546—N3, Alexa 633—Y5). Fluorescent images were acquired in monochrome, and color maps were applied to the images post acquisition. Post hoc linear brightness and contrast adjustment were applied uniformly to the image under analysis.

#### Image analysis

Analysis of mean fluorescence intensities were performed on microscope images without any adjustments, in ImageJ. Specifically, region of interests were marked around mossy fibers in the suprapyramidal layer in CA3 stratum lucidum; the DG granule cell layer; the CA1 stratum radiatum; the CA3 stratum radiatum and the CA1 and CA3 pyramidal cell layer regions. Mean fluorescence intensities were measured using ImageJ. Cell density estimates were based on mean on mean fluorescent intensity from the DAPI for different subregions. For normalization of syt7 measurements, the intensities from mossy fibers were divided with those obtained from the granule cell region—as the granule cell region lacked syt7 expression and the intensities were related to unspecific background fluorescence. For quantification of CB intensities, the same regions were used as for the syt7 for determining mean fluorescence intensity in mossy fiber regions with normalization performed with background intensities from CA3 stratum radiatum region. Mossy fiber convergence ratios were determined by dividing normalized CB intensities in mossy fibers with cell density estimates obtained for CA3 pyramidal cell layer region.

## Supplementary information

Supplementary file 1
