# Supplementary material for: Species-specific differences in synaptic transmission and plasticity
Source: Sci Rep. 2020 Oct 6;10:16557. doi: 10.1038/s41598-020-73547-6 (PMC7538572; doi:10.1038/s41598-020-73547-6)
Supplement: Supplementary file 1 [file 41598_2020_73547_MOESM1_ESM.docx]

**Supplementary Material**

**Species-specific differences in synaptic transmission and plasticity**

**Authors:**

Prateep Beed^1,2^*^‡^, Saikat Ray^3,4^*^‡^, Laura Moreno Velasquez^1^*, Alexander Stumpf^1^*, Daniel Parthier^1^, Aarti Swaminathan^1^, Noam Nitzan^1^, Jörg Breustedt^1^, Liora Las^4^, Michael Brecht^3^ & Dietmar Schmitz^1,2,5,6,7‡^

**Affiliations:**

1 Neuroscience Research Center, Charité - Universitätsmedizin Berlin, Germany

2 Berlin Institute of Health, 10178 Berlin, Germany

3 Bernstein Center for Computational Neuroscience, Humboldt University of Berlin, Philippstr. 13, Haus 6, 10115 Berlin, Germany

4 Department of Neurobiology, Weizmann Institute of Science, 76100 Rehovot, Israel

5 German Center for Neurodegenerative Diseases (DZNE) Berlin, 10117 Berlin, Germany

6 Cluster of Excellence NeuroCure, 10117 Berlin, Germany

7 Einstein Center for Neurosciences Berlin, 10117 Berlin, Germany

* These authors contributed equally

^‡^To whom correspondence should be addressed: prateep.beed@charite.de, saikat.ray@weizmann.ac.il or dietmar.schmitz@charite.de

**List of Supplementary Material:**

1. Figures S1, S2 & S3
2. Table S1

**Supplementary Figures**


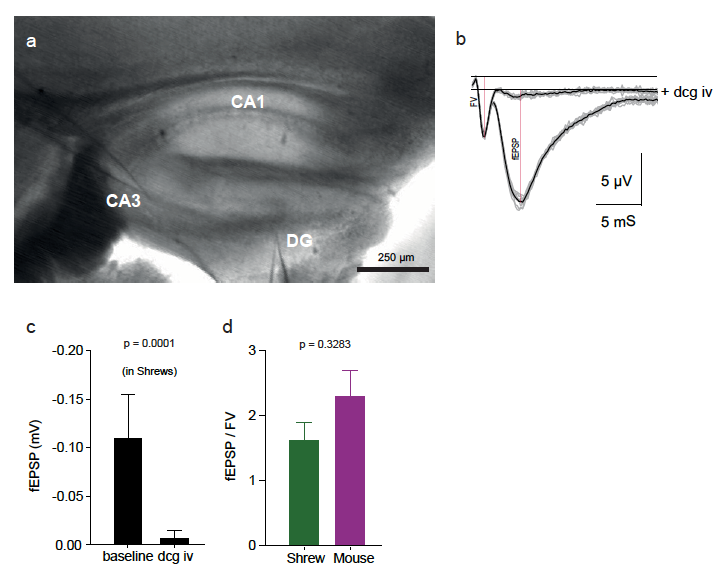


**Figure S1 (related to Fig 2) Mossy fibers in shrews are also DCG IV sensitive similar to mice.**

**(a)** Stimulation electrode were placed in the dentate gyrus while the recording electrode was placed in the stratum lucidum in the CA3 area to record mossy fiber inputs.

**(b-c)** Mossy fiber fEPSPs are DCG IV sensitive **(b)** and the reduction is quantified in **(c)**.

**(d)** fEPSP to fiber volley ratio are not significantly different between shrews (green) and mice (purple)


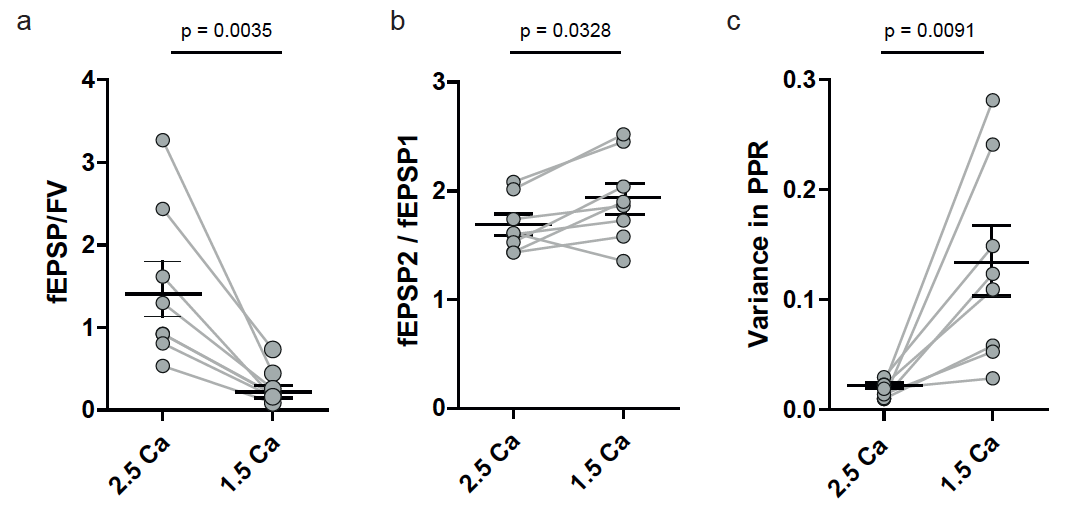


**Figure S2 (related to Fig 2) Paired pulse ratios in shrews are minimally affected by changing extracellular calcium concentration at the mossy fiber synapse.**

**(a)** External calcium was reduced from 2.5mM to 1.5mM while recording mossy fiber fEPSP and an expected reduction in the fEPSP to FV change.

**(b)** Also paired pulse ratio is increased on lowering external calcium.

**(c)** For every experiment the variance was calculated from 20 sweeps in 2.5 and 1.5mM Calcium. As expected the variance in PPR is higher in lower calcium.


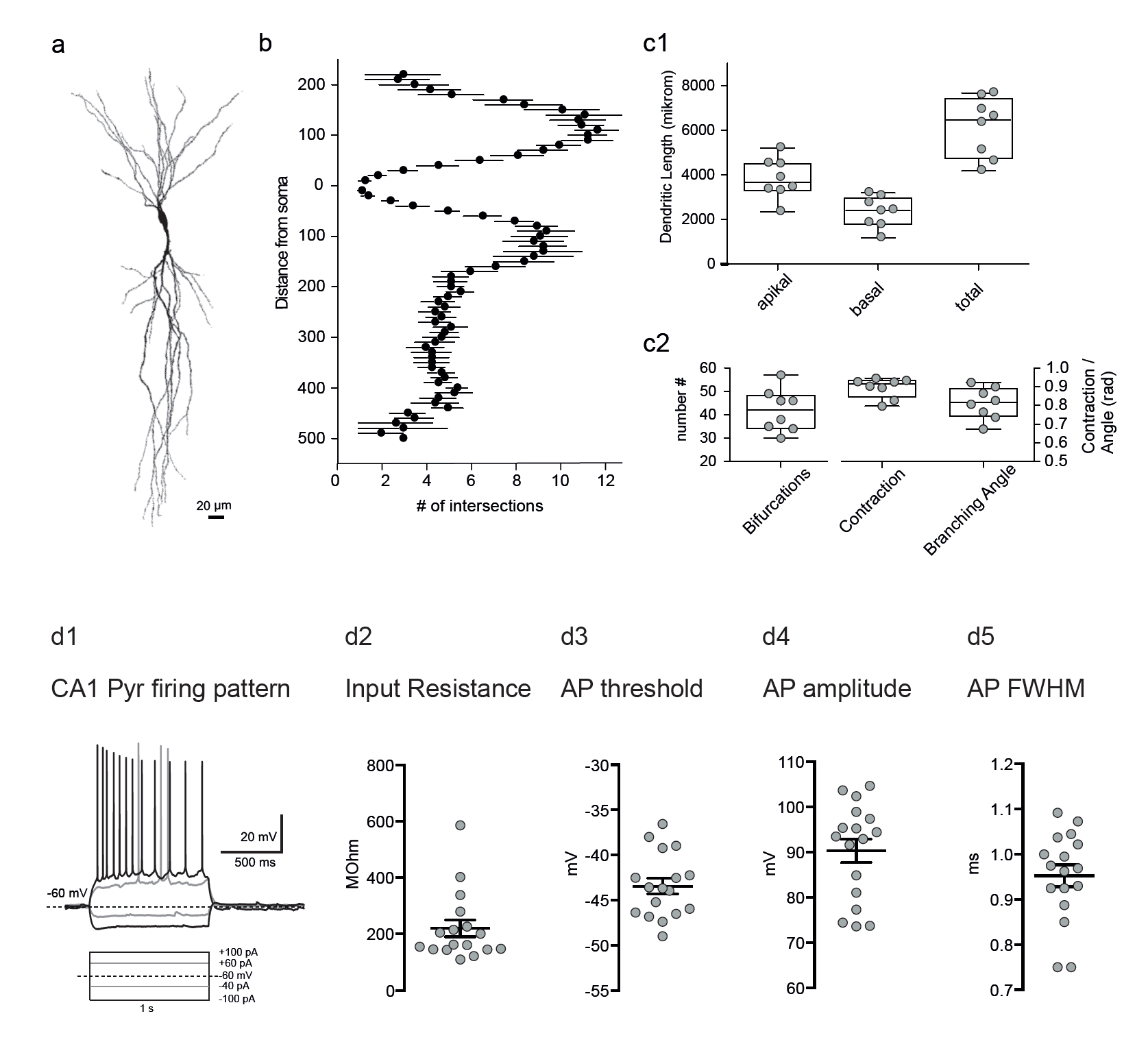


**Figure S3 (related to Fig 5) Conserved microanatomy and cellular physiology of pyramidal cells in CA1 of the shrew.**

**(a)** An example of a biocytin labeled CA1 pyramid

**(b)** Spatial profile of number of intersections of CA1 pyramids in shrews with respect to distance from soma.

**(c1)** Apical, basal and total dendritic length of CA1 pyramids in shrews (apical: 3802 ± 318 µm, basal: 2313 ± 244 µm, total: 6115 ± 472 µm, n= 8 cells)

**(c2)** Bifurcations, contraction and branching angle of CA1 pyramids in shrews (bifurcations: 41.9 ± 3.2, contraction: 0.89 ± 0.02, branching angle: 0.81±0.03 rad, n= 8 cells)

**(d)** Intrinsic properties of CA1 pyramids

**(d1)** Firing pattern of CA1 pyramidal cells in Etruscan Shrew

**(d2)** Input resistance

**(d3)** Action potential threshold

**(d4)** Action potential amplitude

**(d5)** Action potential full-width half-maximum

| **Figure** | **Experiment** | **Number of measurements** | **Number of animals** | **Mean ± SEM** | **p-value** | **Statistical test** |
| --- | --- | --- | --- | --- | --- | --- |
| 1a-b | DAPI intensity (au) | - | 7 shrews, 6 mice | - | - |  |
| 1c panel 1 | DAPI intensity (au) | 21 shrews, 18 mice | 7 shrews, 6 mice | 35.87 ± 1.98 (shrews), 35.36 ± 1.73 (mice) | 0.749 | Mann whitney two tailed |
| 1c panel 2 | DAPI intensity (au) | 21 shrews, 18 mice | 7 shrews, 6 mice | 23.89 ± 1.70 (shrews), 20.79 ± 1.27 (mice) | 0.246 | Mann whitney two tailed |
| 1c panel 3 | DAPI intensity (au) | 21 shrews, 18 mice | 7 shrews, 6 mice | 67.56 ± 3.13 (shrews), 61.18 ± 2.41 (mice) | 0.069 | Mann whitney two tailed |
| 1c panel 4 | DAPI intensity (au) | 21 shrews, 18 mice | 7 shrews, 6 mice | 10.84 ± 1.99 (shrews), 10.27 ± 1.35 (mice) | 0.156 | Mann whitney two tailed |
| 1c panel 5 | DAPI intensity (au) | 21 shrews, 18 mice | 7 shrews, 6 mice | 14.39 ± 1.67 (shrews), 12.10 ± 0.97 (mice) | 0.603 | Mann whitney two tailed |
| 1c panel 6 | DAPI intensity (au) | 21 shrews, 18 mice | 7 shrews, 6 mice | 9.87 ± 1.52 (shrews), 10.85 ± 0.85 (mice) | 0.064 | Mann whitney two tailed |
| 1f-h | CB+DAPI intensities (au) | 21 shrews, 18 mice | 7 shrews, 6 mice | 0.26 ± 0.04 (shrews), 0.15 ± 0.02 (mice) | 0.038 | Mann whitney two tailed |
| 2a1-a3 | MF FF | 15 shrews, 13 mice | 12 shrews,  x mice | 240.9 ± 26.32 (shrews), 634.5 ± 45.71 (mice) | <0.0001 | Mann whitney two tailed |
| 2b1-b2 | MF PPR | 13 shrews, 13 mice | 12 shrews,  x mice | 1.717 ± 0.115 (shrews), 2.687 ± 0.286 (mice) | 0.0002 | Mann whitney two tailed |
| 2c1 | MF PTP | 15 shrews, 13 mice | 12 shrews,  x mice | 2.119 ± 0.165 (shrews), 9.342 ± 1.321 (mice) | <0.0001 | Mann whitney two tailed |
| 2d1-d3 | MF LTP | 9 shrews,  7 mice | x shrews,  x mice | 1.189 ± 0.079 (shrews), 1.759 ± 0.186 (mice) | 0.0164 | Mann whitney two tailed |
| 3 | CB+Syt7 intensities (au) | 21 shrews, 18 mice | 7 shrews, 6 mice | 2.04 ± 0.10 (shrews), 3.13 ± 0.17 (mice) | <0.00001 | Mann whitney two tailed |
| 4b-d | Syt7 intensity (au) | - | 6 mice, 7 shrews, 5 bats | - | - | - |
| 4e | Syt7 intensity (au) | 18 | 6 mice | 3.08 ± 0.15 (CA1), 3.13 ± 0.16 (CA3) | 0.41 | Paired t-test |
| 4f | Syt7 intensity (au) | 21 | 7 shrews | 2.74 ± 0.15 (CA1), 2.05 ± 0.09 (CA3) | <0.0001 | Paired t-test |
| 4g | Syt7 intensity (au) | 15 | 5 bats | 1.57 ± 0.06 (CA1), 1.35 ± 0.03 (CA3) | 0.0009 | Paired t-test |
| 5a | CA1 fEPSP/FV | 14 shrews  17 mice |  | 6.479 ± 1.405 (shrews)  9.115 ± 1.521 (mice) | 0.138 | Mann whitney two tailed |
| 5b | CA1 PPR | 14 shrews |  | Shrew data:  1.532 ± 0.079 (50 ms),  1.292 ± 0.047 (100 ms),  1.112 ± 0.024 (200 ms),  1.001 ± 0.013 (500 ms)  Mouse data is extracted from Wozny et al., 2009 | - | - |
| 5c | CA1 LTP | 13 shrews  14 mice |  |  | - | - |
| S1c | MF dcg iv | 14 shrews | 12 shrews | -0.1104 ± 0.044 mV (EPSP), -0.0081 ± 0.007 mV (dcg iv) | 0.0001 | Wilcoxon matched-pairs test |
| S1d | MF fEPSP/FV | 11 shrews,  7 mice | 12 shrews | ­­­1.623 ± 0.266 (shrews)  2.296 ± 0.3978 (mice) | 0.3283 | Mann whitney two tailed |
| S2a | MF fEPSP/FV in low Ca^+2^ | 8 shrews |  | 1.476 ± 0.331 (2.5Ca), 0.279 ± 0.075 (1.5Ca) | 0.0035 | Paired t-test |
| S2b | MF PPR in low Ca^+2^ | 8 shrews |  | 1.680 ± 0.088 (2.5Ca), 1.930 ± 0.142 (1.5Ca) | 0.0328 | Paired t-test |
| S2c | MF PPR Variance in low Ca^+2^ | 8 shrews |  | 0.017 ± 0.003 (2.5Ca), 0.130 ± 0.032 (1.5Ca) | 0.0091 | Paired t-test |
| S3c1 | CA1 morphology | 8 shrews |  | apical: 3802 ± 318 µm, basal: 2313 ± 244 µm, total: 6115 ± 472 µm | - | - |
| S3c2 | CA1 morphology | 8 shrews |  | bif: 41.9 ± 3.2,  contract: 0.89 ± 0.02, br.angle: 0.81 ± 0.03 rad | - | - |
| S3d2 | CA1 Input Resistance | 17 CA1 pyramids | 5 shrews | 220.3 ± 29.7 MΩ | - | - |
| S3d3 | CA1 AP Threshold | 17 CA1 pyramids | 5 shrews | -43.44 ± 0.865 mV | - | - |
| S3d4 | CA1 AP Amplitude | 17 CA1 pyramids | 5 shrews | 90.27 ± 2.597 mV | - | - |
| S3d5 | CA1 AP FWHM | 17 CA1 pyramids | 5 shrews | 0.952 ± 0.024 ms | - | - |

**Table S1. Experiments and respective statistical analysis**
